# Molecular Evolution of the *Vacuolar Iron Transporter* (*VIT*) Family Genes in 14 Plant Species

**DOI:** 10.3390/genes10020144

**Published:** 2019-02-14

**Authors:** Jun Cao

**Affiliations:** Institute of Life Sciences, Jiangsu University, Zhenjiang 212013, Jiangsu, China; cjinfor@163.com

**Keywords:** vacuolar iron transporter, evolution, iron stress, expression, soybean

## Abstract

The vacuolar iron transporter (VIT) proteins are involved in the storage and transport of iron. However, the evolution of this gene family in plants is unknown. In this study, I first identified 114 *VIT* genes in 14 plant species and classified these genes into seven groups by phylogenetic analysis. Conserved gene organization and motif distribution implied conserved function in each group. I also found that tandem duplication, segmental duplication and transposition contributed to the expansion of this gene family. Additionally, several positive selection sites were identified. Divergent expression patterns of soybean *VIT* genes were further investigated in different development stages and under iron stress. Functional network analysis exhibited 211 physical or functional interactions. The results will provide the basis for further functional studies of the *VIT* genes in plants.

## 1. Introduction

As an essential microelement, iron (Fe) is involved in several important cellular processes in plants, including photosynthesis, nitrogen fixation, respiration, and DNA and hormone synthesis [1]. Although iron is abundant in the Earth’s crust, most iron is present in the insoluble ferric form, which is not available for biological purposes [2,3]. To overcome this barrier, plants have evolved two strategies (I and II) for iron acquisition. Strategy I plants include most non-graminaceous monocots and dicots that first secrete protons for the acidification of the rhizosphere [4,5], and then reduce Fe^3+^ to Fe^2+^ using root ferric-chelate reductase 2 (FRO2) [6], and finally uptake of Fe^2+^ across the root plasma membrane using the iron-regulated transporter 1 (IRT1) [7,8]. Strategy II plants are all graminaceous monocots. These plants first release Fe^3+^-specific phytosiderophores (PS) to form Fe^3+^-PS chelate complex, and then absorb this complex into root cells via the yellow stripe 1 transporter [9,10].

After entering the cells, iron is tightly regulated to avoid excessive accumulation and cause cytotoxicity [11]. At the cellular level, iron is either incorporated into proteins or stored in specific cell compartments. Ferritin is a major protein complex with 24 subunits, which can store up to 4500 Fe atoms per molecule in the bioavailable form [12,13]. In addition, vacuolar compartmentalization or sequestration can also be exploited to regulate iron homeostasis [14]. Vacuolar iron transporters (VITs) were found to play significant roles in this process. Under high iron environment, *VITs* can maintain iron in the optimal physiological range and prevent cellular toxicity. For example, in *Arabidopsis*, the AtVIT1 protein can transport iron into vacuoles for normal seedling development when exposed to high iron conditions [15]. AtMEB1 and AtMEB2 also serve as iron transporters to reduce toxicity of the yeast *ccc1* mutant under high iron condition [16]. Additionally, some *Arabidopsis VIT1*-like proteins (named AtVTL1, AtVTL2, and AtVTL5) have been shown to contribute to the regulation of iron homeostasis in plants [17]. *OsVIT1* and *OsVIT2* are highly expressed in the flag leaf blades and sheaths of rice, and plants with mutations in these genes showed reduced iron content in flag leaves, suggesting a role for OsVIT1 and OsVIT2 in regulating vacuolar iron transport [18]. Recently, over-expression of the *Brassica napus* vacuolar iron transporter (*BnMEB2*) was found to enhance the tolerance of iron toxicity in transgenic *Arabidopsis* plants [19]. Due to its role in iron storage, VIT is potentially good candidate for iron biofortification [20]. In addition, some *VIT* orthologs from *Tulipa gesnariana* and *Centaurea cyanus* also regulate flower colors by mediating the iron transport into the vacuoles of petals [21,22]. These observations suggest that these important iron transporters can regulate various physiological processes of plants.

Although quite a few *VITs* have been functionally characterized in the model plant *Arabidopsis*, rice, and others, the functions of most members of this gene family remain unknown. Moreover, no study has explored the evolutionary relationships of the *VIT* gene family in plants. The new availability of some model plant genomes facilitates the evolution study of *VIT* gene family. Here, 114 *VIT* genes were first identified from 14 plant species, and integrated evolutionary analysis was then performed.

## 2. Materials and Methods

### 2.1. Identification of the *Vacuolar Iron Transporter* Genes from 14 Plant Species

To identify potential VIT genes in 14 plant genomes (*Arabidopsis thaliana*; *Brachypodium distachyon; Chlamydomonas reinhardtii*; *Cucumis sativus; Glycine max; Medicago truncatula*; *Oryza sativa*; *Physcomitrella patens*; *Populus trichocarpa*; *Selaginella moellendorffii*; *Solanum lycopersicum*; *Sorghum bicolor*; *Vitis vinifera*; *Zea mays*), the HMM (Hidden Markov Model) profile of the VIT domain (PF01988) obtained from the Pfam database (http://pfam.xfam.org/) [23] was used as queries to perform BLAST searches in the phytozome database (http://www.phytozome.net) [24] with -1 E threshold. Furthermore, all 251 candidate proteins identified by the domain blast were used for new queries in this database. Finally, SMART [25] and Pfam [23] were further used to confirm the obtained sequences.

### 2.2. Estimation of *Vacuolar Iron Transporter* Gene Gain and Loss in 14 Plant Species

To determine the degree of variation in the number of *VIT* family genes in the 14 plant species, clades were divided based on the phylogeny. Notung v 2.6 [26] was used to infer gene gain and loss events after reconciling the gene tree and the species tree for each clade.

### 2.3. Phylogeny, Gene Organization, and Conserved Motif Analysis of the *Vacuolar Iron Transporter* Gene Family

The MUSCLE method in MEGA6 [27] was used to perform multiple sequence alignment of all predicted proteins. Next, phylogenetic trees using neighbor-joining (NJ) with a *p*-distance substitution model, 1000 bootstrap replications and pairwise deletion gaps and maximum likelihood (ML) methods with Jones-Taylor-Thornton (JTT) model, 100 bootstrap replicatons and partial deletion were constructed, respectively. By comparing the *VIT* genomic sequences and coding sequences (CDS) from the phytozome database (http://www.phytozome.net) [24], the gene organization information was inferred. In addition, the conserved motifs were identified using the MEME program [28] with the following parameters: zero or one motif in each sequence, 6 and 50 width of motifs, and a maximum of 8 motifs.

### 2.4. Estimation of *Vacuolar Iron Transporter* Gene Duplication Pattern in Tomato and Soybean

The chromosomal localization of *VIT* genes was determined based on the annotation of the phytozome database (http://www.phytozome.net) [24]. The duplication patterns of *VIT* genes were investigated in tomato, soybean and other plants. Tandem duplication was defined if putative paralogues were either adjacent or separated by less than five genes in the same chromosome. Segmental duplication was considered if paralogues were located in the known genomic duplication blocks [29,30]. Plant Genome Duplication Database (PGGD, http://chibbaagtecugaedu/duplication/) [31] was used to determine if the VITs were present in the genomic duplication blocks. Given similar synonymous substitution rates (*Ks*) within duplicated genes, the *Ks* value was used as the proxy for time. I first used the K-Estimator 6.0 program [32] to estimate the *Ks* values of paralogous genes. And then the timing of duplication events was calculated using the formula (T = *Ks*/2λ) with previously described clock-like rates (λ) of λ = 15 × 10^−8^ for tomato and soybean [33].

### 2.5. Site-Specific Selection Assessment and Prediction of Protein Secondary Structure

The *K_a_/K_s_* value was used to estimate selective pressure by calculating the synonymous rate (*K_s_*) and the non-synonymous rate (*K_a_*) at each codon. In this study, I used a Bayesian inference approach [34] to calculate site-specific positive and purifying selection. Three evolutionary models [M8 (β + w>=1), M7 (β), and M5 (γ)] were used to describe how the characteristics evolve in probabilistic terms. The best model-fitting biological assumption was selected. Briefly, a statistical distribution was firstly assumed to account for heterogeneous *K_a_/K_s_* values among sites. Next, eight discrete categories were used to approximate the distributions. Finally, the *K_a_/K_s_* values were computed by calculating the expectation of a posterior distribution [34]. The prediction of protein (Glyma.20G166100) secondary structure was performed using the Protter tool (http://wlab.ethz.ch/protter/start/) with a custom protein sequence in FASTA format and other defaults [35]. To investigate the difference of selective pressures on different amino acid residues during evolution, these sites were marked with different colors.

### 2.6. Expression Analysis of the Soybean *Vacuolar Iron Transporter* Gene Family based on RNA-Seq Data

RNA-seq data available from the phytozome database (http://www.phytozome.net) [24] was used to investigate the expression profiles of *VIT* genes in nine different tissues. The values of FPKM (fragments per kilobase of exon per million mapped reads) were calculated as log10 values. The expression data were normalized and viewed using the Genesis (v 1.76) program [36].

### 2.7. Soybean Sample Preparation, RNA Isolation, Quantitative Real-Time PCR (qRT-PCR)

Soybean “zhonghuang35” seedlings were grown in liquid MS media in a greenhouse at 24 °C temperature with a 14 h photoperiod. Two-week-old seedlings were exposed to MS liquid solution adding 0.2mM FeSO_4_·7H_2_O (pH5.5) and MS liquid solution (pH 5.5) for iron stress and mock treatments, respectively. Total RNA was extracted from the whole seedlings after 12 h and 24 h treatment using the TRIzol^®^ total RNA extraction kit (Sangon). RNase free DNase-I was used to remove genomic DNA. M-MLV (TakaRa) was used to perform reverse transcription, followed by quantitative assays of each diluted cDNA sample using an ABI 7500 sequence detection system. The mean of three experiments stands for their relative expression levels. Eight soybean *VIT* genes were subjected to qRT-PCR analysis using the primers listed in Table S1. The soybean *actin* gene (*Glyma.18G290800*) was used as the endogenous control. Finally, the 2^−∆∆CT^ method [37] was used to calculate the relative expression level of the *VIT* genes.

### 2.8. Network Assembly

Protein–protein interaction networks were assembled using the STRING database (http://string-db.org) [38]. This database includes some interaction sources, such as textmining, experiments, databases, co-expression, neighborhood, fusion, co-occurrence and so on [38]. All predicted soybean *VIT* proteins were submitted to this database. Minimum required interaction score was set as medium confidence (0.400). Max number of interactors was shown no more than 10 on both the first and the second shell.

## 3. Results

### 3.1. Identification and Distribution of *Vacuolar Iron Transporter* Genes in Plants

To identify the putative *VIT* family genes from the 14 plant species. I first performed a BLAST search of the phytozome database (http://www.phytozome.net) [24] described above. From this search, 114 putative *VIT* genes were identified. The number of *VIT* genes in each species ranged from two in *Chlamydomonas reinhardtii* to 21 *VIT* genes in the soybean genome. These wide range suggested that the number of *VIT* genes changed in different plants during evolution. To better understand how the *VIT* gene family evolved in the 14 plants species, the number of *VIT* genes in the most recent common ancestor (MRCA) was estimated. Reconciliation of the species phylogeny with the gene trees using Notung software [26] suggested that a single ancestral *VIT* gene existing in the MRCA of these plant species. An additional four genes were obtained before the appearance of these species (Figure S1). Altogether, five ancestral *VIT* genes were detected in the ancestor of plants, with one retained ancestral gene and four that were lost when the Chlamydomonas lineage appeared. After inheriting the ancient *VIT* genes from its Embryophyte ancestor, these genes were subsequently duplicated and lost before the Angiosperm divergence. In this period, two gain and six loss events resulted in an overall decrease in the number of *VITs* from the land plants (*P. patens*) to the angiosperms. Eudicot ancestral *VITs* expanded over three times after the separation from the monocot ancestor, which also expanded over two times about 145 million years ago [39]. About 19 and 13 ancestral *VIT* genes were identified in the MRCA of eudicots and monocots, respectively. After that, many *VITs* were lost in the eudicots and monocots. Of the eudicots, only soybean increased the number of genes in the *VIT* family. Compared with the number in eudicots and monocots, the size of the *VIT* family was reduced in all analyzed species. For example, the number of *VITs* decreased approximately 30.8, 46.2, and 68.4 percent for maize, rice, and *Arabidopsis*, respectively. The estimated numbers of genes in the MRCA of Viridiplantae were five (Figure S1). Compared with the number of ancestral *VIT* genes, this family expanded in most detected species except moss and Chlamydomonas.

### 3.2. Phylogenetic Analysis, Gene Organization, and Conserved Motifs Distribution

**Figure 1:**
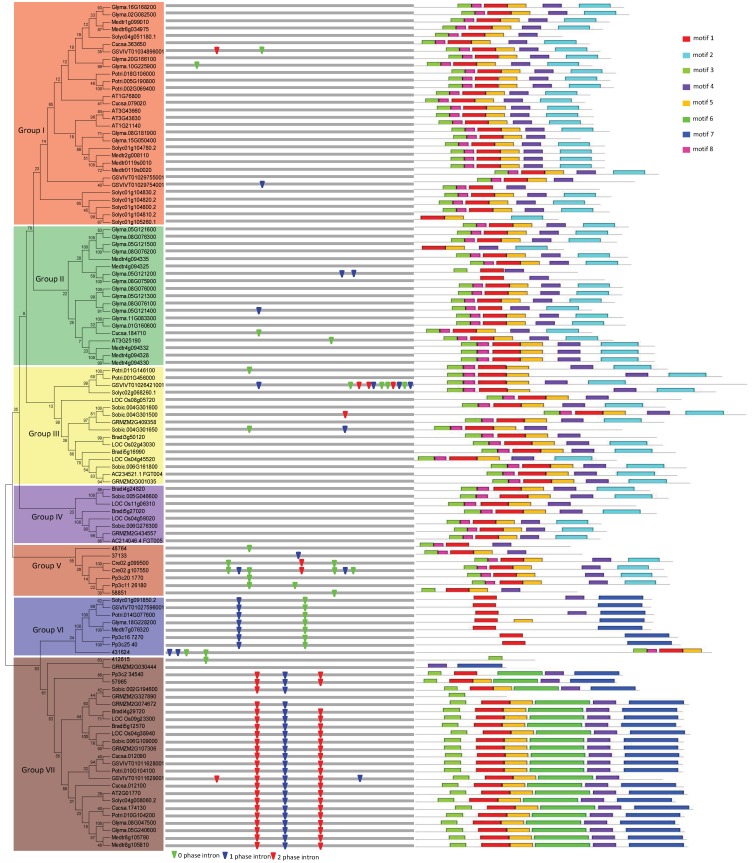
Phylogenetic relationship, gene structure and motif composition of the *vacuolar iron transporter* (*VIT*) genes in fourteen plant species. The phylogenetic tree is constructed and classified into seven major groups (from Group I to Group VII). The insertion positions of 0, 1, and 2 phase introns are marked with green, blue, and red inverted triangles, respectively. Different motifs of the *VIT* proteins are displayed by different colored boxes.

To assess the evolutionary relationship of these *VIT* genes in the 14 plant species, phylogenetic analysis of VIT proteins was performed based on NJ and ML methods with MEGA6 [27]. The phylogenetic trees from the NJ and ML methods have a very similar topology. Here, I choose the NJ tree for further analysis. The 114 predicted VIT proteins were classified into seven groups based on sequence similarity, from Group I to Group VII (Figure 1). Other evidence, such as gene organization and conserved motifs distribution, is described below and also supports this classification. Group I is the largest with 30 members, representing 26.3% of the total number of *VIT* genes, and Groups V is the smallest, with only seven genes. In addition, some eudicot-specific *VIT* clades formed Groups I, II, and VI, and some monocot-specific *VIT* clades formed Group IV (Figure S2). These members appeared only after the monocot and eudicot separation, suggesting potential specific roles for eudicots or monocots. The Group V only contains *VIT* genes from fern, moss, and green alga. *VITs* in Group VII are present in all detected embryophytes.

Intron gain and loss is a common phenomenon in evolution, which can increase the complexity of gene organization [40,41]. To further examine the organizational diversity of *VIT* genes, the exon-intron structure was investigated through compared the genomic sequences with the coding sequences. A detailed illustration of gene organization, containing the distribution, position, and phase of introns, is shown in Figure 1. In general, *VIT* gene organization was well conserved, especially in Groups V, VI, and VII, supporting a common origin in each group. I found that there are no introns in Group IV, and a few introns are scattered in other groups. Intron loss may be the consequence of duplication and intron retention. In this study, I found one event of intron retention. *Glyma.10G225900* and *Glyma.20G166100* derive from a common ancestor (Figure 1). Further sequence analysis indicated that after duplication, one intron was removed in the transcript of *Glyma.10G225900*, but the same intron was retained in the transcripts of *Glyma.20G166100.* I further used this retained amino acid residues to perform BLAST searching in other plants. The result indicated that this intron retention event also occurred in the *Potri002G069400* and *Potri005G190800* genes in the poplar, and other *VIT* genes in other species, such as *Phvul007G079100* in *Phaseolus vulgaris*; *SapurV1A0066s0090* and *SapurV1A0066s0100* in *Salix purpurea*.

To further examine the diversification of VIT proteins, I identified their conserved motifs using MEME [28]. As a result, eight conserved motifs were found in the predicted VIT proteins (Figure 1; Table 1). The similar motif compositions of each group not only provided additional evidence supporting the phylogenetic analyses, but also implied functional relevance. Some distinct motifs were also found in specific groups. For instance, motif six is restricted in Group VII. Future studies should examine the function of the distinctive motifs. From another point of view, the different motif composition also implies functional diversification among different groups. Additionally, the differences in VIT sequences of different groups may also increase the complexity of the function.

### 3.3. Duplication Events of the *Vacuolar Iron Transporter* Genes

**Figure 2:**
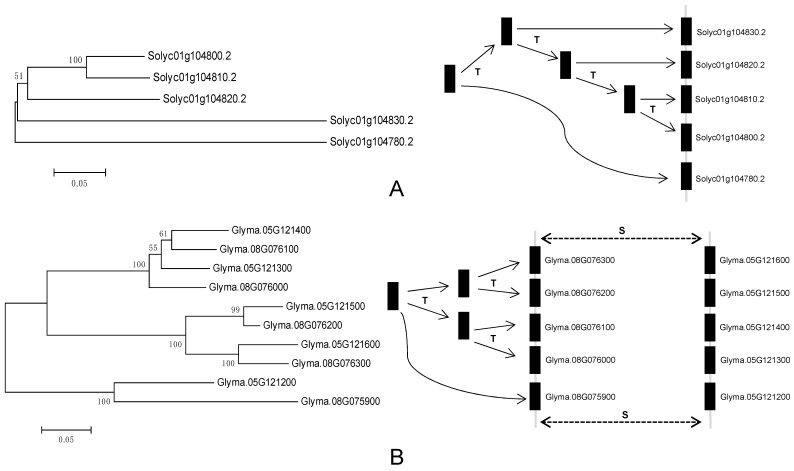
Origins of some tomato and soybean *VIT* genes by tandem and segmental duplication. The letters T and S indicate the positions where tandem and segmental duplication have occurred, respectively.

To further investigate the relationship between genetic divergence and gene duplication, I determined the chromosomal location of each *VIT* gene as described above using the annotation in the phytozome database (http://www.phytozome.net). There are relatively more *VIT* genes in soybean and tomato, which is the basis of analysis of duplication events. Therefore, I mainly investigated the duplication characteristics in the two species. The results indicated that *VIT* genes are distributed unevenly among chromosomes three and ten of the tomato and soybean genome, respectively. About 70 percent of tomato *VIT* genes localized to chromosome 1. For soybean, over 61.9 percent of *VIT* genes are in chromosomes 5 and 8, and the vast majority of *VIT* genes are present in tandem. A diagram was used to describe the evolutionary relationships of these genes (Figure 2). Tomato *Solyc01g1047802* gene was first duplicated and produced the *Solyc01g1048302* gene, and then the *Solyc01g1048302*, after three tandem duplications, produced three other homologous genes (Figure 2). A similar process occurred in the *VIT* cluster region of chromosome 8 in soybean. Unlike the *VIT* cluster of the tomato chromosome 1, a segmental duplication occurred again in the soybean chromosome 8 *VIT* cluster, forming another *VIT* cluster region in soybean chromosome 5 (Figure 2). Overall, tandem and segmental duplications were the major factors contributing to the expansion of the *VIT* gene family in tomato and soybean. To better understand the evolutionary history of the *VIT* family, *Ks* values were used as the proxy for time to estimate the timing of these duplication events (Table 2). The *Ks* values of tomato *VIT* parologues ranged from 0.34785 to 0.78947, suggesting that the tandem duplication events occurred about 11.59–26.32 million years ago (Mya) on tomato chromosome 1. This date was approximately in line with the recent large-scale duplication event of tomato genome [33]. I also found that the initial tandem duplication (*Glyma.08G075900*–*Glyma.08G076300*) occurred about 41.78 Mya on the *VIT* cluster region of soybean chromosome 8. After about 20 million years, the second tandem duplication (*Glyma.08G075900*–*Glyma.08G076100*) occurred when soybean and alfalfa lineages were separated from their common ancestor. After that, another tandem and segmental duplications occurred about 2.93–6.61 Mya, close to the recent large-scale duplication event (polyploidy) of the soybean genome [33,42]. In addition, I also analyzed the duplication patterns of *VIT* genes in other plant species and found that tandem duplication and transposition can contribute to the increase in the number of *VIT* genes in these species (Figure S3).

### 3.4. Selective Pressure Analysis among Different Amino Acid Sites

**Figure 3:**
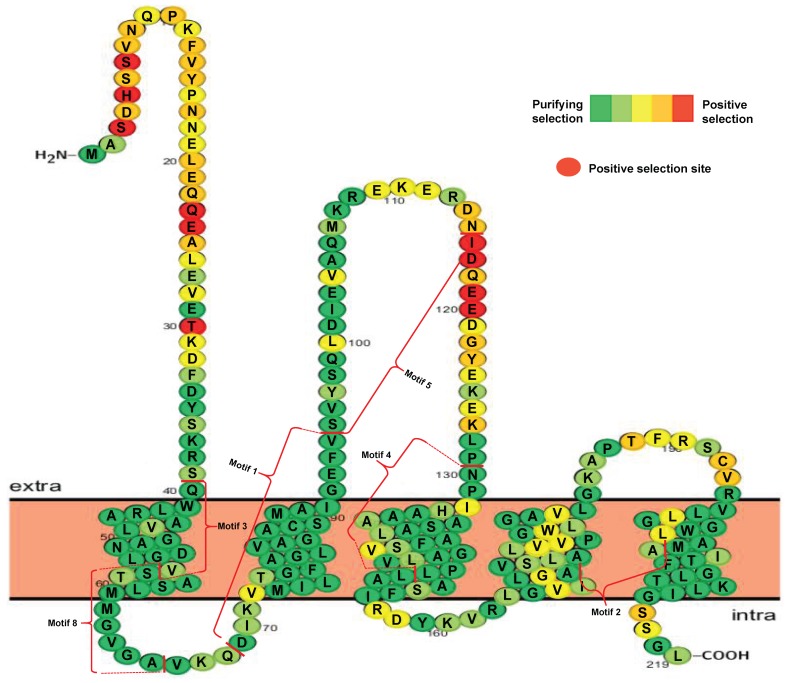
Distribution of positive selection sites of VIT members in Group I predicted by M8 model.

Phylogenetic results suggested that seven groups were generated after *VIT* duplication in the 14 plant species. To explore which amino acid substitution was subjected to selective pressure after duplication, I further investigated variable selective pressures among different VIT sites in each group. The results indicated that the *K_a_/K_s_* values differed for each group (Table S2). The *K_a_/K_s_* values are relatively higher in Groups II, III, and VI than the values for the other groups, indicating a faster changing rate (Table S2). Despite these differences, all the *K_a_/K_s_* values are lower than 1, suggesting that the most VIT proteins are under purifying selection in evolution. However, some positive selection sites, such as VIT members in Groups I, II, III, and VII predicted by the M8 model and Groups II, III, and VI predicted by the M5 model, were also found in this analysis (Table S2). However, the M7 selection model did not indicate the presence of any positively selected sites. As an example, the detailed distribution of the different selection sites in Group I sequences predicted by the M8 model is shown in Figure 3. Ten amino acid sites were found to be under positive selection. Of these, six positive sites were located in the N-terminal portion, and the other four sites were located in the extra membrane loop region (Figure 3).

### 3.5. Divergent Expression Profiles of the Soybean *Vacuolar Iron Transporter* Genes in Different Tissues and under Iron Stress

**Figure 4:**
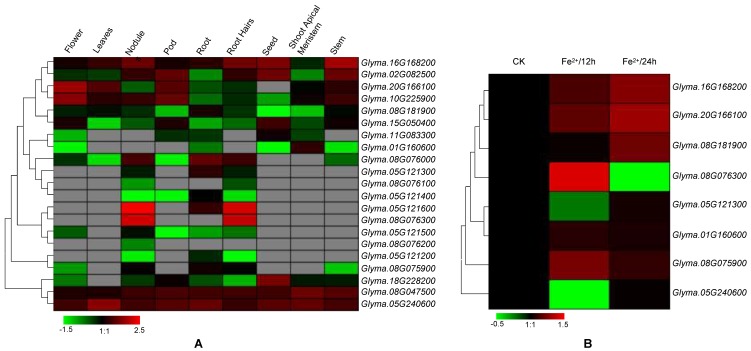
Expression patterns of the *VIT* genes in soybean different developmental stages and under iron stress by RNA-seq data (**A**) and qRT-PCR (quantitative real time polymerase chain reaction) (**B**), respectively. Heat maps reflect the strength of relative expression.

To understand the roles of specific *VIT* genes in different tissues, I next investigated the expression profiles of soybean *VIT* genes using available RNA-seq data. Transcription profiles of the *VIT* genes were collected and analyzed in nine different tissues (Figure 4A). The results revealed that the *VIT* genes showed diverse expression profiles in these different tissues, suggesting different roles for a variety of developmental stages. Some *VIT* genes were significantly abundant in some tissues. For instance, *Glyma.05G12160* and *Glyma.08G07630* transcripts accumulated more in the nodule and root hair stages than in the other tissues. The highest level of *Glyma.20G166100* and *Glyma.10G225900* gene transcripts are found in the flower stage, whereas the expression levels of the *Glyma.16G168200* and *Glyma.02G082500* were highest in the stem development stage.

To further determine the involvement of soybean *VIT* genes in response to iron stress, I next analyzed their expression patterns under iron stress by qRT-PCR (Figure 4B). Eight genes were randomly selected. The expression levels of *Glyma.16G168200*, *Glyma.20G166100*, and *Glyma08G075900* were induced at both 12 h and 24 h after iron treatment. Transcript of the *Glyma08G076300* gene was induced at 12 h of iron stress and was suppressed at 24 h of iron stress, indicating that the expression of this gene was controlled by iron stress at different time. The expression of *Glyma.05G121300*, *Glyma.01G160600* and *Glyma.05G240600* remained at a similar level between mock and 24 h iron treatment. These results suggested that soybean *VIT* genes are involved in the response to iron stress. In addition, I investigated the functional divergence of the duplicated genes. Interestingly, for the nine pairs of duplicated *VIT* genes, all paired genes did not exhibit similar expression patterns. For examples, *Glyma05G240600* showed much higher expression in leaf tissue than the expression of its paralogous gene *Glyma.08G047500*. *Glyma.16G168200* expressed to higher levels than *Glyma.02G082500* in all development stages except the pod tissue.

### 3.6. Network Analysis of the Soybean *Vacuolar Iron Transporter* Members

**Figure 5:**
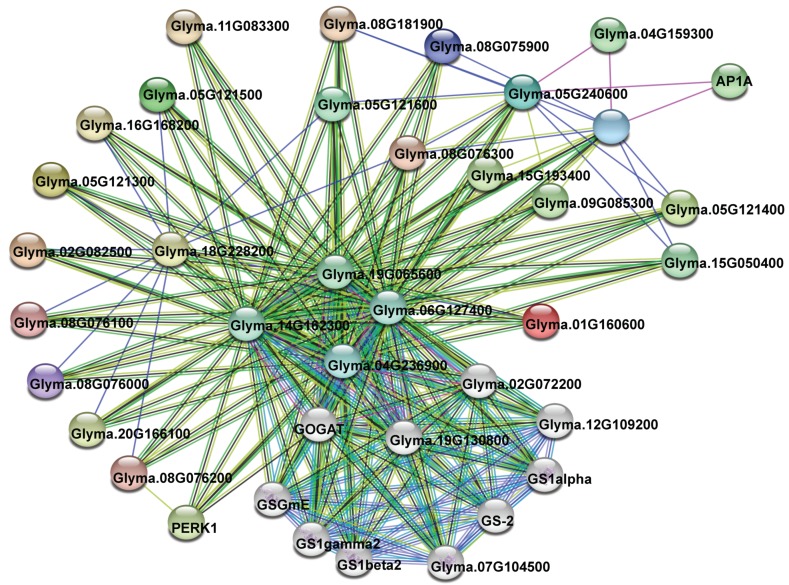
Functional network assembly of the soybean *VIT* genes. 211 interactions were exhibited among a total of 37 genes. Colored nodes mean query *VIT* proteins and first shell of interactors, and white nodes stand for the second shell of interactors. Light blue and purple lines represent the known interactions from cured databases and experimentally determined, respectively. Green, red, and blue lines stand for the predicted interactions from gene neighborhood, fusions, and co-occurrence, respectively. In addition, Light green, black, and dark blue lines represent other interactions from textmining, co-expression, and protein homology, respectively.

To further understand which proteins are potentially interacted with each other by members of *VIT* family, I assembled a protein interaction network using the STRING database [38]. The network was based on some experimental or predicted interactions. As a result, 19 of 21 soybean *VIT* members appeared in the network, exhibiting 211 interactions by a total of 37 unique genes (Table S3). Among them, 21 interactions occurred between the *VIT* proteins, and one *VIT* (Glyma.18G228200) could interact with ten other members (Figure 5; Table S3). Some glutamate synthases (GOGATs), glutamine synthetases (GSs), MADS-box proteins, cation cotransporters, and proline-rich receptor-like protein kinases (PERKs) were predicted as the main interaction partners of soybean *VITs*, which will be discussed below in detail.

## 4. Discussion

In this study, 114 putative *VIT* genes were first identified in 14 plant species, which were further classified into seven groups. Intron retention is a distinct mechanism of gene expression control [43]. Intron retention occurs when an intron is preserved in the final mature mRNA, as it is not excised by the action of the spliceosome. Usually, this retained intron contains premature termination codons that will be removed by the nonsense-mediated decay (NMD) mechanism [44]. My study found that one intron retention event occurred in the transcripts of the *Glyma.20G166100* gene. Moreover, I found that the intron retention also occurred in the *VIT* gene of other plant species, such as *Potri002G069400* and *Potri005G190800* genes in poplar, *Phvul007G079100* in *P. vulgaris*, *SapurV1A0066s0090* and *SapurV1A0066s0100* in *S. purpurea*, indicating conservation in these species. Therefore, as a source of sequence variability, intron retention might regulate VIT protein isoform production to increase the complexity of this protein family.

Genomic complexity is attributed to the duplication, loss or rearrangement of large numbers of genes or chromosomal segments [45]. According to the DDC (duplication-degeneration-complementation) model, duplicated genes are usually faced with three possible fates: non-functionalization, neo-functionalization, and sub-functionalization [46]. Tandem duplication, segmental duplication and transposition are major forces in genomic evolution. Some studies have indicated that gene duplication or loss may be used as a factor that potentially changes the genetic composition of the genome and contributes to the species evolution [47]. Duplication and divergence of MADS-box genes are considered as the main contributors to the evolution of plant floral morphology [48,49]. A duplicated KNOX (KNOTTED1-LIKE HOMEOBOX) transcription factor regulates leaf shape in plants [50]. Three *CBF* (*C-REPEAT-BINDING FACTOR*) genes control plant cold tolerance, and loss of one *CBF* results in reduced cold tolerance in a specific ecological region, suggesting the importance of duplicated genes in influencing species range due to abiotic constraints [51]. In addition, some duplicated oil biosynthesis genes tend to be retained in soybean and *Brassica napus* [52,53]. Therefore, paralogous genes generated by these duplication events formed gene families and played key roles in development, signaling, and stress response [54]. In this study, most *VIT* genes are the results of tandem duplication, segmental duplication and transposition in these plant genomes, suggesting they are major factors responsible for this gene family expansion and may contribute to some functional divergence.

The detection of positively selected amino acid sites is essential for insight into protein structure and function [55]. Previous studies indicated that sub-functionalized genes are usually under purifying selection, and neo-functionalized genes are under positive selection during evolution [33,56]. My study also identified several positive selection sites located at different protein positions (Figure 3). It suggests that these variable regions might play important roles in the binding and transporting of iron. Strikingly, all positive selection sites mapped to the variable regions of *VIT* proteins, implying that these sites might increase the function divergence of these proteins. In addition, expression profiles can provide some insights into the potential functions of genes [57]. As described above, tandem and segmental duplications occurred in the *VIT* locus of soybean (Figure 2). Of eight selected *VIT* genes, three (*Glyma.05G121300*, *Glyma.08G076300*, and *Glyma.08G075900*) were derived from this locus, originating from the same ancestral gene. I also found that the response patterns of these three genes to iron stress were completely different from each other. All of these findings suggested that functional divergence has occurred among these duplicated genes, and the products of these genes might play different roles in soybean development or in iron stress response. Some studies have reported different transcription patterns occurred of duplicated genes [58,59]. In my study, the diverse expression profile of the *VIT* genes might be the result of sub-functionalization or neo-functionalization processes in soybean.

During the long process of evolution, plants have developed a series of strategies, such as ferritin binding and vacuolar sequestration, to counter iron stress. Among them, only 5% of iron is bound by ferritin proteins, and the main location for iron storage is vacuole [60]. Several plant *VITs*, such as *AtVIT1*, *AtVTL1*, *AtVTL2*, *AtVTL5*, *OsVIT1*, *OsVIT2*, and *TaVIT2* are involved in the iron storage process of vacuolar sequestration to regulate plant iron homeostasis [15,17,18,20]. In addition to plants, this unique transporter family also exist in fungi and protists such as *Plasmodium*, but they are absent from metazoans [61]. Yeast has evolved complex mechanisms to obtain iron and to protect itself from the toxic effect of excess cellular iron. This process is mediated with a *CCC1* (*Ca^2+^-SENSITIVE CROSS-COMPLEMENTER*) protein, which can transport iron into vacuoles under iron stress [62]. Recently studies have shown that *Plasmodium VIT* also performs a similar function of removing excess iron from the cytoplasm and preventing iron toxicity with plant *VIT* and yeast CCC1 proteins [61,63]. Therefore, this family genes play similar roles in the evolution of plant, yeast and some protists.

Genes involved in related biological processes are usually cooperatively expressed and function together. In my network analysis, some proteins were predicted as the main interaction partners of soybean *VIT* members (Figure 5; Table S3). Nitrogen is a necessary nutrient for plant growth and development. Through the GS/GOGAT cycle, inorganic nitrogen is assimilated into amide residue of glutamine in all plants [64]. GS catalyzes the conversion of glutamate into glutamine, while GOGAT catalyzes the transfer of an amide group from glutamine to 2-oxoglutarate (2-OG) to produce two molecules of glutamate [65,66]. Several studies have demonstrated that over-expression or mutation of these genes affects the normal growth and development of plants [67,68,69]. In this study, I found that some GOGATs, such as Glyma.19G065600, Glyma.14G162300, and Glyma.19G130800, were predicted to interact with the highest number of soybean VIT proteins. Among the 19 soybean *VIT* proteins existing in the network, about 94.7 percent (18 members) and 78.9 percent (15 members) potentially interacted with Glyma.19G065600 and Glyma.14G162300, respectively (Figure 5; Table S3). It suggests that *VIT* proteins may be involved in the nitrogen metabolism and ammonium assimilation in soybean. MADS-box transcription factors are an ancient family of genes. Many MADS-box genes have been characterized to play key roles in a variety of plant developmental processes, such as determination of flowering time [70], fruit ripening [71], embryo development [72], and resistance abiotic stress [73]. I observed that one MADS-box transcription factor (AP1A, Glyma.06G205800) was predicted to interact with soybean VIT proteins (Glyma.05G240600 and Glyma.08G047500), implying that the AP1A may regulate the transcription of these *VIT* genes, and that they may play a coordinating role in the plant development. Through coupled to potassium or sodium irons, cation cotransporters mediate electroneutral translocation of chloride ions [74]. In animals, cation cotransporters have a variety of functions, including salt transport, neuronal development, and cell volume regulation [75,76]. To date, plant cation cotransporters have been cloned and functionally characterized from *Arabidopsis*, rice and grapevine. Knockout of these genes resulted in severe growth and developmental phenotypes [76,77,78]. Interestingly, in my network analysis, two soybean cation cotransporters (Glyma.09G085300 and Glyma.15G193400) were observed to interact with the *VIT* proteins (Glyma.05G240600 and Glyma.08G047500), respectively (Figure 5; Table S3), implying that the soybean *VIT* proteins may also participate in electroneutral translocation of other ions. Furthermore, as a small group of receptor-like kinases, PERKs are thought to act as sensors [79]. During cell expansion or stresses, PERKs can monitor changes of the cell wall and activate the associated cellular responses [80]. In my study, soybean PERK1 protein (Glyma.09G191300) was also predicted to interact with one *VIT* protein (Glyma.08G076200) (Figure 5; Table S3). These results suggested the diversity of *VIT* binding proteins, which were helpful to understand the function roles of the *VITs* in various metabolisms.

In conclusion, this study provided a systemic analysis of the *VIT* gene family in plants. The gene family had a birth process in plant evolution. Gene organization and motif compositions are highly conserved, indicative of their functional conservation. *VIT* genes are non-randomly distributed across the genome, and most members derived from tandem, segmental duplications and transposition. Several amino acid sites were positively selected during evolution. Expression patterns also indicated functional divergence for the soybean *VIT* genes. Functional network analyses identified some potential related genes. Overall, my results provide the basis for further functional study of this important gene family.

## Figures and Tables

**Table 1:** Conserved motifs identified by Multiple Em for Motif Elicitation (MEME) among plant *VIT* proteins.

Motif	Width	Sites	E-Value	Sequences
1	26	110	1.5e-1588	DSKAMJLAGFAGLVAGACSMAIGEFV
2	35	66	2.1e-1225	VSLALAVFGGLGAVLGKAPVVRSCLRVLIGGWLAM
3	15	102	3.3e-987	QWLRAAVLGANDGLV
4	21	104	1.9e-1023	NPLQAAAASALAFSVGALVPL
5	21	98	2.8e-873	SVYSQYDIEVAZMKREQEEID
6	50	23	3.8e-863	PDTEAAEVAEILSQYGJEPHEYGPVVNALRKNPQAWLDFMMKFELGLEKP
7	50	30	1.5e-674	VLASVVVTLLALLIFGYAKGRFTGNRPFLSAVQTALIGAJASAAAYGMAK
8	11	75	1.5e-423	STASLMMGVGA

**Table 2:** Divergence time of the *VIT* paralogues in tomato and soybean.

Species	Gene 1	Gene 2	*Ks*	Divergence Time (Mya)	Duplication Types
*Solanum lycopersicum*	*Solyc01g1047802*	*Solyc01g1048302*	0.78947	26.32	tandem duplication
	*Solyc01g1048302*	*Solyc01g1048202*	0.61314	20.44	tandem duplication
	*Solyc01g1048202*	*Solyc01g1048102*	0.52749	17.58	tandem duplication
	*Solyc01g1048102*	*Solyc01g1048002*	0.34785	11.59	tandem duplication
	*Solyc01g0918502*	*Solyc04g0080602*	0.87754	29.25	transposition
*Glycine max*	*Glyma.08G075900*	*Glyma.08G076300*	1.25353	41.78	tandem duplication
	*Glyma.08G075900*	*Glyma.08G076100*	0.65802	21.93	tandem duplication
	*Glyma.08G076300*	*Glyma.08G076200*	0.16939	5.65	tandem duplication
	*Glyma.08G076100*	*Glyma.08G076000*	0.18062	6.02	tandem duplication
	*Glyma.08G076300*	*Glyma.05G121600*	0.14334	4.78	segmental duplication
	*Glyma.08G076200*	*Glyma.05G121500*	0.08789	2.93	segmental duplication
	*Glyma.08G076100*	*Glyma.05G121400*	0.16184	5.39	segmental duplication
	*Glyma.08G076000*	*Glyma.05G121300*	0.1751	5.84	segmental duplication
	*Glyma.08G075900*	*Glyma.05G121200*	0.19831	6.61	segmental duplication
	*Glyma.08G047500*	*Glyma.05G240600*	0.10616	3.54	transposition
	*Glyma.08G181900*	*Glyma.15G050400*	0.34178	11.39	transposition
	*Glyma.20G166100*	*Glyma.10G225900*	0.12651	4.22	transposition
	*Glyma.16G168200*	*Glyma.02G082500*	0.09795	3.27	transposition

## Supplementary Materials

The following are available online at https://www.mdpi.com/2073-4425/10/2/144/s1.
