# Supplementary material for: Molecular Evolution of the Vacuolar Iron Transporter (VIT) Family Genes in 14 Plant Species
Source: Genes (Basel). 2019 Feb 14;10(2):144. doi: 10.3390/genes10020144 (PMC6409731; doi:10.3390/genes10020144)
Supplement: Supplementary file 1 [file genes-10-00144-s001.zip › supplentary data/Fig. S2.pdf]

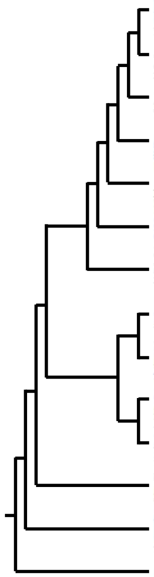

	Group I	Group II	Group III	Group IV	Group V	Group VI	Group VII	Total
G. max	6	12				1	2	**21**
M. truncatula	5	5				1	2	**13**
C. sativus	2	1					3	**6**
P. trichocarpa	3		2			1	2	**8**
A. thaliana	4	1					1	**6**
V. vinifera	3		1			1	2	**7**
S. lycopersicum	7		1			1	1	**10**
O. sativa			3	2			2	**7**
B. distachyon			2	2			2	**6**
Z. mays			3	2			4	**9**
S. bicolor			4	2			2	**8**
S. moellenforffii					3	1	2	**6**
P. patens					2	2	1	**5**
C. reinhardtii					2			**2**
**Total**	**30**	**19**	**16**	**8**	**7**	**8**	**26**	
