# Supplementary figures and images for: Molecular Evolution of the Vacuolar Iron Transporter (VIT) Family Genes in 14 Plant Species
Source: Genes (Basel). 2019 Feb 14;10(2):144. doi: 10.3390/genes10020144 (PMC6409731; doi:10.3390/genes10020144)

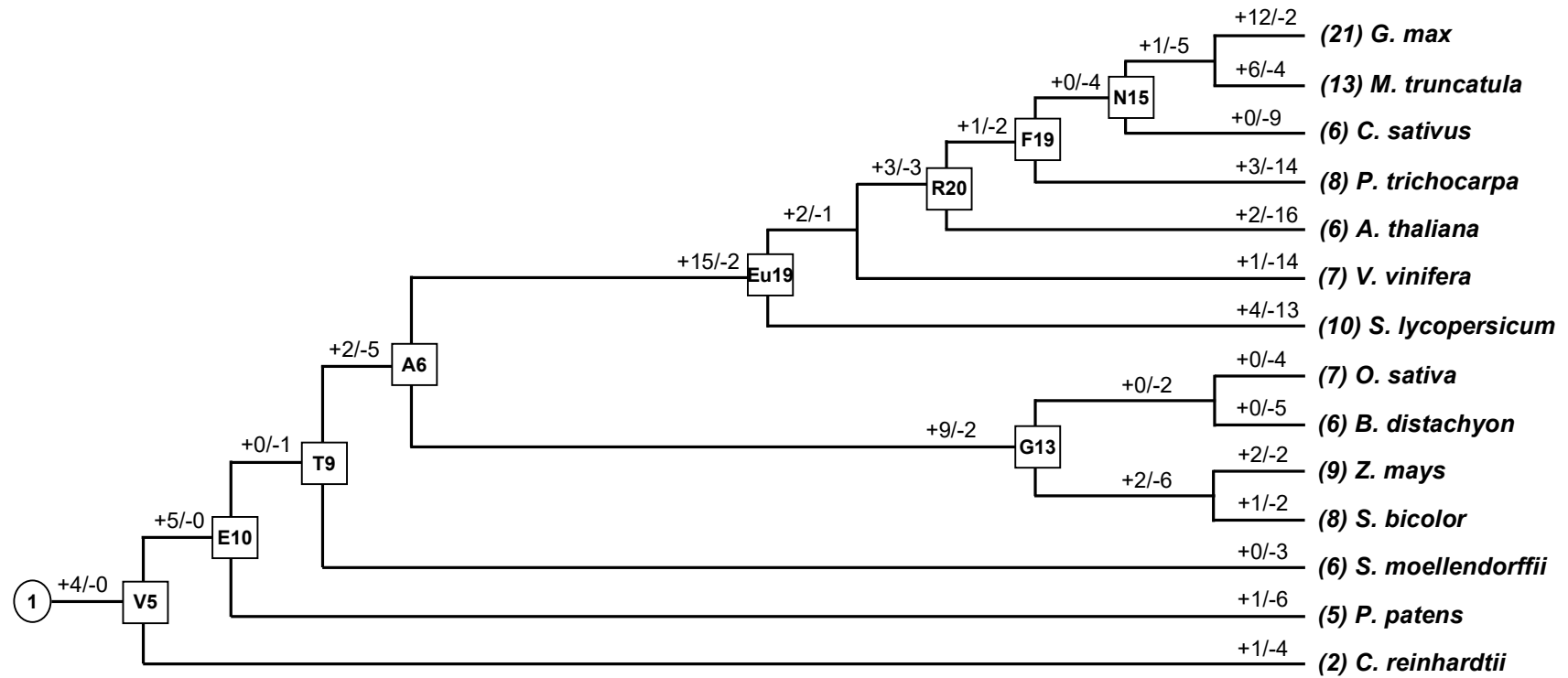

Supplement: Supplementary file 1 [file genes-10-00144-s001.zip › supplentary data/Fig. S1.pdf]

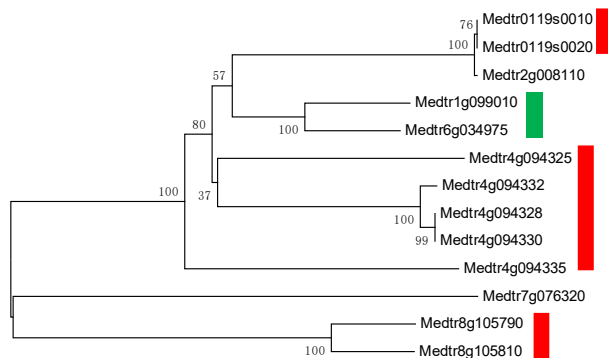

0.05

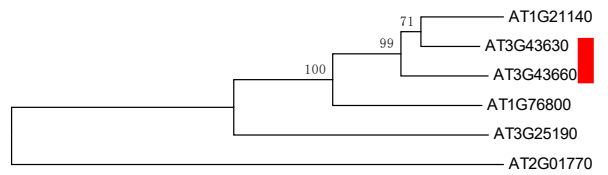

0.05

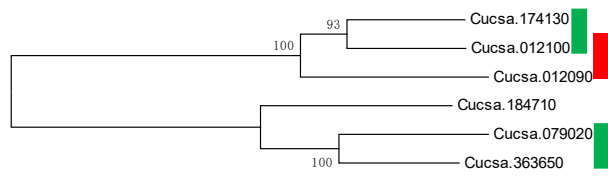

0.05

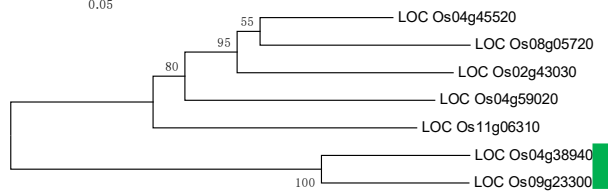

0.1

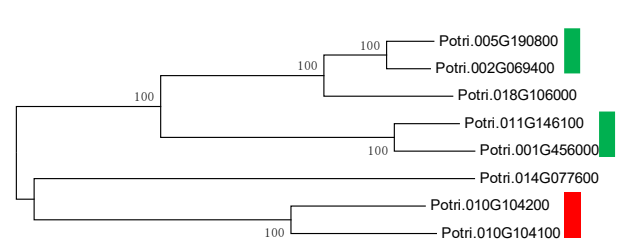

0.1

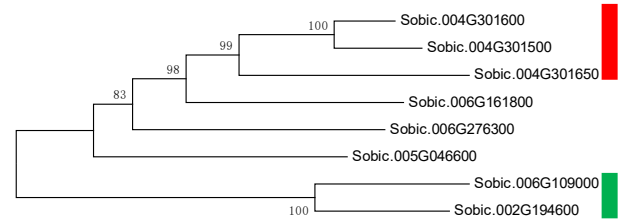

0.1

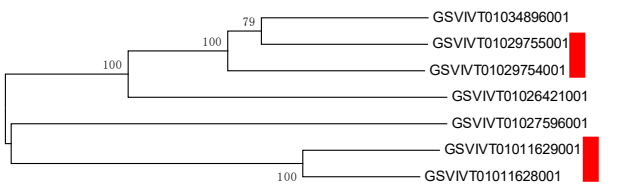

0.1

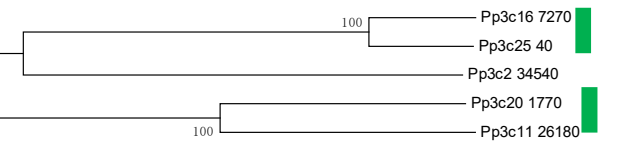

0.1

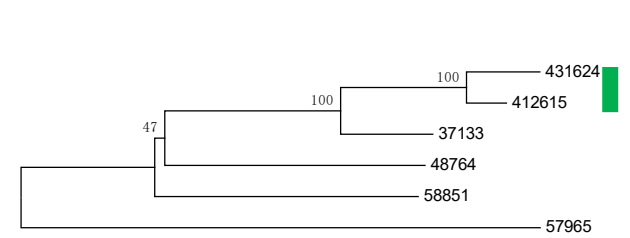

0.1

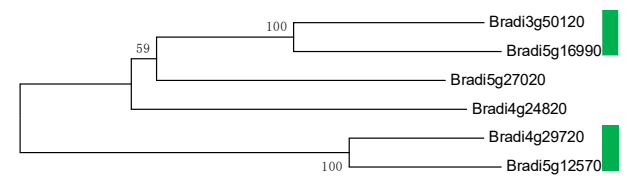

0.05

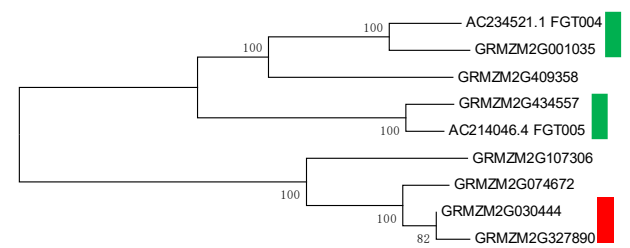

0.05

tandem duplication  
transposition

Supplement: Supplementary file 1 [file genes-10-00144-s001.zip › supplentary data/Fig. S3.pdf]
