# Supplementary material for: Molecular Evolution of the Vacuolar Iron Transporter (VIT) Family Genes in 14 Plant Species
Source: Genes (Basel). 2019 Feb 14;10(2):144. doi: 10.3390/genes10020144 (PMC6409731; doi:10.3390/genes10020144)
Supplement: Supplementary file 1 [file genes-10-00144-s001.zip › supplentary data/Table S1.doc]

Table S1: Primers used in this study.

| **Primer names** | **Primer sequences (5`-3`)** | **Usage** |
| --- | --- | --- |
| actin-F | CTTGACTGAGCGTGGTTAT | qRT-PCR for reference gene |
| actin-R | GCTGGTCCTGGCTGTCT |  |
| Glyma.16G168200-F | GTCATCAAATCAAGCAT | qRT-PCR |
| Glyma.16G168200-R | GTAGTCAAAGGTGTCCTCA |  |
| Glyma.20G166100-F | GGTGAGGCTTGGTGTGA | qRT-PCR |
| Glyma.20G166100-R | GACCCAATCAACTTGGTTA |  |
| Glyma.05G121300-F | TGGAGAGTTTGTATCTGT | qRT-PCR |
| Glyma.05G121300-R | GCTGAAGCTAGTGCAGCATGA |  |
| Glyma.08G076300-F | GCATTGGTGCCTCTACT | qRT-PCR |
| Glyma.08G076300-R | CCAAGAAGGAACCTTACA |  |
| Glyma.08G075900-F | CATATTGAGATTCTCATCA | qRT-PCR |
| Glyma.08G075900-R | GCTCCAAACACAGCTCCA |  |
| Glyma.08G181900-F | CTGTGAGGAAAGATGTTA | qRT-PCR |
| Glyma.08G181900-R | GTTCGGCAACTTATCCTT |  |
| Glyma.01G160600-F | GAAGTTGCCAAATCCATT | qRT-PCR |
| Glyma.01G160600-R | ACTGCTCCAACACCACCA |  |
| Glyma.05G240600-F | GCCACCGAACACAGACTA | qRT-PCR |
| Glyma.05G240600-R | CAATGGAGGAGGTGGCAT |  |
| GmVIT1-F | TCTAGAATGGCAAACAACAATATTCA | Over-expression |
| GmVIT1-R | CCCGGGAGCTACTCCAAGTCCAAGATGA |  |

*Restrictive endonucleases sites (*Xba*I and *Sma*I) were added in the primer GmVIT1-F and GmVIT1-R to facilitate vector construction, respectively.
