# Supplementary material for: Molecular Evolution of the Vacuolar Iron Transporter (VIT) Family Genes in 14 Plant Species
Source: Genes (Basel). 2019 Feb 14;10(2):144. doi: 10.3390/genes10020144 (PMC6409731; doi:10.3390/genes10020144)
Supplement: Supplementary file 1 [file genes-10-00144-s001.zip › supplentary data/Table S2.doc]

Table S2: Likelihood values and parameter estimates of the *VIT* genes.

| **Branches** | **Models** | ***Ka/Ks*** | **Log-likelihood** | **Numbers of PSS*** |
| --- | --- | --- | --- | --- |
| Group I | M8(beta+w>=1) | 0.2991 | -11297.1 | 10 |
|  | M7(beta) | 0.2692 | -11303.1 | 0 |
|  | M5(gamma) | 0.292 | -11293.2 | 0 |
| Group II | M8(beta+w>=1) | 0.4243 | -7145.26 | 32 |
|  | M7(beta) | 0.3537 | -7159.69 | 0 |
|  | M5(gamma) | 0.4203 | -7145.74 | 29 |
| Group III | M8(beta+w>=1) | 0.5553 | -9824.75 | 60 |
|  | M7(beta) | 0.4439 | -9838.54 | 0 |
|  | M5(gamma) | 0.5884 | -9826.55 | 55 |
| Group IV | M8(beta+w>=1) | 0.1867 | -3548.09 | 0 |
|  | M7(beta) | 0.1882 | -3548.97 | 0 |
|  | M5(gamma) | 0.1867 | -3547.91 | 0 |
| Group V | M8(beta+w>=1) | 0.2193 | -4846.21 | 0 |
|  | M7(beta) | 0.2164 | -4847.45 | 0 |
|  | M5(gamma) | 0.2621 | -4849.21 | 0 |
| Group VI | M8(beta+w>=1) | 0.3662 | -6291.08 | 0 |
|  | M7(beta) | 0.3505 | -6290.77 | 0 |
|  | M5(gamma) | 0.4242 | -6310.05 | 3 |
| Group VII | M8(beta+w>=1) | 0.2231 | -10571.9 | 12 |
|  | M7(beta) | 0.1886 | -10593.6 | 0 |
|  | M5(gamma) | 0.2032 | -10582.8 | 0 |

*PSS: positive selection site
